# An organogenesis network-based comparative transcriptome analysis for understanding early human development *in vivo *and *in vitro*

**DOI:** 10.1186/1752-0509-5-108

**Published:** 2011-07-06

**Authors:** Hai Fang, Wen Jin, Ying Yang, Ying Jin, Ji Zhang, Kankan Wang

**Affiliations:** 1 State Key Laboratory of Medical Genomics, Sino-French Research Center for Life Sciences and Genomics, Ruijin Hospital affiliated to Shanghai Jiao Tong University School of Medicine (SJTU-SM), Ruijin Rd. II, Shanghai 200025, China; 2 Key Laboratory of Stem Cell Biology, Institute of Health Sciences, Shanghai Institutes for Biological Sciences, Chinese Academy of Sciences and SJTU-SM, 225 South Chongqing Road, Shanghai 200025, China; 3 Department of Computer Science, University of Bristol, Woodland Road, Bristol BS8 1UB, UK; 4 Shanghai Stem Cell Institute, 225 South Chongqing Road, Shanghai 200025, China

**Keywords:** Integrated networks, Human organogenesis, Stemness-relevant module, Differentiation-relevant module, Gene set enrichment analysis, Transcriptome

## Abstract

**Background:**

Integrated networks hold great promise in a variety of contexts. In a recent study, we have combined expression and interaction data to identify a putative network underlying early human organogenesis that contains two modules, the stemness-relevant module (hStemModule) and the differentiation-relevant module (hDiffModule). However, owing to its hypothetical nature, it remains unclear whether this network allows for comparative transcriptome analysis to advance our understanding of early human development, both *in vivo *and *in vitro*.

**Results:**

Based on this integrated network, we here report comparisons with the context-dependent transcriptome data from a variety of sources. By viewing the network and its two modules as gene sets and conducting gene set enrichment analysis, we demonstrate the network's utility as a quantitative monitor of the stem potential *versus *the differentiation potential. During early human organogenesis, the hStemModule reflects the generality of a gradual loss of the stem potential. The hDiffModule indicates the stage-specific differentiation potential and is therefore not suitable for depicting an extended developmental window. Processing of cultured cells of different types further revealed that the hStemModule is a general indicator that distinguishes different cell types in terms of their stem potential. In contrast, the hDiffModule cannot distinguish between differentiated cells of different types but is able to predict differences in the differentiation potential of pluripotent cells of different origins. We also observed a significant positive correlation between each of these two modules and early embryoid bodies (EBs), which are used as *in vitro *differentiation models. Despite this, the network-oriented comparisons showed considerable differences between the developing embryos and the EBs that were cultured *in vitro *over time to try to mimic *in vivo *processes.

**Conclusions:**

We strongly recommend the use of these two modules either when pluripotent cell types of different origins are involved or when the comparisons made are constrained to the in *vivo *embryos during early human organogenesis (and an equivalent *in vitro *differentiation models). Network-based comparative transcriptome analysis will contribute to an increase in knowledge about human embryogenesis, particularly when only transcriptome data are currently available. These advances will add an extra dimension to network applications.

## Background

Molecular and genetic interaction networks have proven to be useful in a variety of contexts. They can potentially be used to predict gene functions [1], to predict perturbation phenotypes [2] and genetic modifier loci [3], to identify human disease genes and drug targets [4], to increase the statistical power in human genetics [5,6], and to study pathogen/virus-host crosstalk [7,8], to name just a few examples. Typically, they are constructed through the integration of multiple data sources such as expression data and interaction data [9-12]. The motivations for building such networks include the following: (i) from a biological perspective, genes are assumed to be interconnected into cohesive networks that control a certain biological process and (ii) from a methodological perspective, the integration of multiple layers of information is more likely to identify biologically relevant signals than analysis of either data source alone. Therefore, these integrated networks hold great promise for explaining the control mechanisms that underlie particular physiological and developmental processes.

In humans, embryogenesis is a complex process that consists of several sequential developmental events: fertilization, blastulation, gastrulation, and organogenesis [13]. Although several studies have attempted to understand the molecular networks that control early embryogenesis (the oocyte and preimplantation stages) [14-18], the extent to which these developmental events can be explained by their underlying networks is still unknown. The molecular profiling of human organogenesis is increasingly becoming the focus of considerable research [19-21]. Recently, we have reported the first comprehensive transcriptome analysis of early organogenesis, which ranged from Carnegie stages 9 (S9) to 14 (S14) [20]. Through the in-depth data mining [22-24] and comparisons with mouse embryos [25] and human embryonic stem cells (hESCs) [26-28], we have found sets of genes that are important for the initiation and maintenance of early human organogenesis. With further integration of interaction data [29-34], we have also shown that the coordination of early human organogenesis is probably under the control of a shared molecular network, or a human organogenesis network (hORGNet; see Additional File 1). Preliminary analysis has revealed that this network contains a stemness-relevant module (hStemModule) and a differentiation-relevant module (hDiffModule). Given the hypothetical nature of this network [19,20], additional research is warranted to further explore its potentials for characterizing early human organogenesis. It also remains unclear whether this network can be extended to describe the other stages of human organogenesis. Because the network is inherently associated with two modules, there is a great need to clarify the circumstances in which it can be used as a reference for evaluating the stem potential *versus *the differentiation potential.

To do this, we started with our previously identified network (i.e., the hORGNet and its two modules, hStemModule and hDiffModule) [20]. The network itself is associated with the intrinsic features of expression information from early human organogenesis and well-curated interaction information from existing human interactome resources. The genes in this network are collectively informative as a molecular signature of this developmental window, similar to the concept of using disease-perturbed networks as a basis for understanding disease initiation and progression [35]. With this network at hand, we applied gene set enrichment analysis (GSEA) to perform expression-based inspections of the hORGNet and its two modules in different, yet representative developmental contexts, including human organogenesis, various human stem cell types, and a hESC-derived embryoid body (EB) model. These comparisons demonstrate the ability of this integrated network to improve our coarse-grained understanding of early human development, both *in vivo *and *in vitro*.

## Results

### The comparative transcriptome analysis pipeline using an integrated network during early human organogenesis (hORGNet)

**Figure 1:**
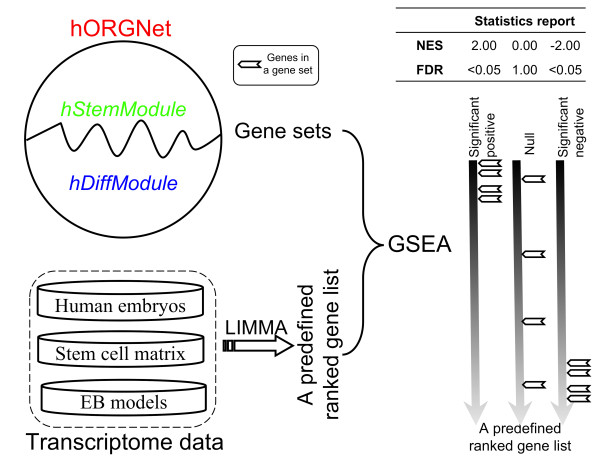
**Schematic flowchart illustrating the network-oriented comparisons using transcriptome data in a variety of developmental contexts**. Gene set enrichment analysis (GSEA) takes the hORGNet (and its two modules, hStemModule and hDiffModule) as a gene set and determines the degree to which genes in the gene set are overrepresented at the top or bottom of a ranked gene list. The ranked gene lists are predefined by a LIMMA supervised analysis of three representative context-specific transcriptome datasets, including human embryos, stem cell matrix, and EB models. The right panel illustrates the hypothetical results of GSEA analysis. Genes in a gene set tend to be at the top ("Significant positive"), at the bottom ("Significant negative") or randomly distributed ("Null") over a predefined ranked gene list. The normalized enrichment score (NES) reflects the degree to which genes in a gene set are overrepresented at the top or bottom of the ranked gene list. A positive NES (e.g., 2.00) indicates overrepresentation at the top of the ranked gene list, whereas a negative NES (e.g., -2.00) indicates overrepresentation at the bottom. The significance of the overrepresentation corresponding to each NES can be assessed by false discovery rate (FDR).

The procedures for network-orientated comparisons are illustrated in Figure 1. Briefly, transcriptome data from a variety of developmental contexts are available from public databases such as NCBI GEO [36]. In this study, we focused on three representative developmental contexts, including human embryos [20,21], the stem cell matrix (a transcriptome dataset of various human stem cell phenotypes [37]), and EB models [38,39]. By viewing the genes in the hORGNet collectively as a signature (or gene set) of early human organogenesis, we were able to apply GSEA analysis [40] to explore the possibility of using the hORGNet to re-interpret these context-specific transcriptome data. To do this, we first ranked the gene lists based on Linear Models for Microarray Data (LIMMA) supervised analysis of these context-specific transcriptome data [41]. Next, we performed GSEA analysis to determine the degree to which genes in the hORGNet (and its two modules, hStemModule and hDiffModule) were overrepresented at the top or bottom of the ranked list of genes. We used this rank-based comparative approach because it has been proven to be highly reproducible and interpretable [42]. GSEA reports several useful statistics for interpreting the results, including a normalized enrichment score (NES) and a false discovery rate (FDR) [40]. The former indicates a positive or negative correlation, while the latter indicates the statistical significance. By analyzing transcriptome data from the stem cell matrix, EB models and human embryos, we found that the hORGNet and its two modules can advance our understanding of early human development, both *in vivo *and *in vitro*.

### The two modules of the hORGNet capture the expression patterns of early human organogenesis

**Figure 2:**
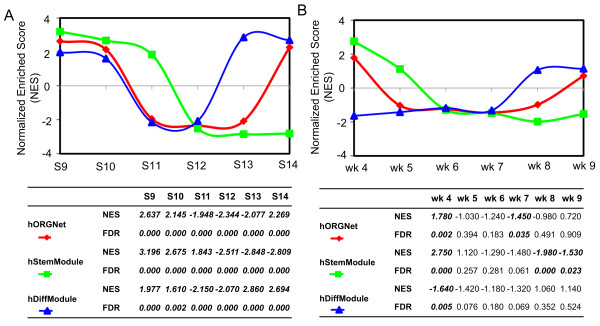
**GSEA of the hORGNet and its two modules (hStemModule and hDiffModule) using transcriptome data during human organogenesis**. **(A) **Human embryos from Carnegie stages 9 (S9) to 14 (S14). **(B) **Human embryos during weeks (wk) 4-9. The normalized enrichment score (NES) reflects the degree to which the hORGNet (in red), the hStemModule (in green) and the hDiffModule (in blue) are overrepresented at the top (reflected by a positive NES) or bottom (indicated by a negative NES) of a ranked gene list predefined by expression data from each stage. Statistically significant NES enrichments (i.e., FDR < 0.05) of NES are highlighted with bold and italic text.

Previously, we have constructed a hORGNet based on both expression and interaction information [20]. Preliminary analysis indicates that the hORGNet is probably inherited with the Yin-Yang crosstalk of a stemness-relevant module (hStemModule) and another differentiation-relevant module (hDiffModule). Prior to the applications to other developmental processes, we first asked whether the hORGNet and its two modules were associated with the gradual loss of the stem potential and the increased diversity of the differentiation potential during development. To address this question, we conducted GSEA analysis of the hORGNet and its two modules using transcriptome data of human embryos from Carnegie stages 9 (S9) to 14 (S14) [20]. The GSEA results showed that the hStemModule enrichments monotonically shifted from the most positive at S9 (NES = 3.196; FDR = 0) to the most negative at S14 (NES = -2.809; FDR = 0), whereas the hDiffModule showed more dynamic changes during early human organogenesis (Figure 2A). Recently, another study has reported a transcriptome analysis of human embryos during weeks (wk) 4-9 [21]. As shown in Figure 2B, the GSEA results showed that the hStemModule enrichments decreased gradually from a significant positive correlation at wk 4 (NES = 2.750; FDR = 0) to no significant correlation during wk 5-7 (FDR > 0.05) to a significant negative correlation at wk 8 (NES = -1.980; FDR = 0) and at wk 9 (NES = -1.530; FDR = 0.023). A significant negative correlation between the hDiffModule and wk 4 human embryos was also observed (NES = -1.640; FDR = 0.005). Beyond wk 4 (i.e., out of the developmental window S9-S14), however, we found there was no significant correlation between the hDiffModule and human embryos (see the bottom panel in Figure 2B). Taken together, analysis of this developmental window (S9-S14) of early human organogenesis and the beyond suggests that the hStemModule may in general reflect the gradual loss of the stem potential, while the hDiffModule reflects the dynamic changes in the differentiation potential that are required for proper differentiation at each stage of this developmental window.

### The hStemModule is a general indicator that distinguishes different cell types in terms of the stem potential

Stem cell matrix is a database for transcriptome data from various cultured cells including pluripotent, multipotent and differentiated cell types [37] (for details see Methods, and shown in Additional File 2). We first used the stem cell matrix to test whether the hStemModule is indicative of the stem potential in these varied cell types. As shown in Table 1 (also see Additional File 3), the application of GSEA to the stem cell matrix showed that the hStemModule was significantly and positively correlated with pluripotent cells including embryonic pluripotent stem cells (ePSC; NES = 2.132; FDR = 0), teratocarcinoma pluripotent stem cells (tPSC; NES = 1.899; FDR = 0) and induced pluripotent stem cells (iPSC; NES = 2.658; FDR = 0). In contrast, no correlation or negative correlation was observed with other multipotent and differentiated cell types (Table 1). Furthermore, the iPSCs were more likely to be associated with the hStemModule than either the ePSCs or the tPSCs (Table 2 and Additional File 4; see Discussion). More surprisingly, the hStemModule was also positively correlated with embryonic pluripotent stem cell-derived embryoid bodies (ePSC_EB; NES = 2.020; FDR = 0) (Table 1). To exclude the possibility of artifacts associated with the above observation, we chose another set of transcriptome data from an early stage EB (3.5 days) that was derived from two hESC lines (H1 and H9) [38] as an independent validation. The GSEA results again indicated that there was a significant positive correlation between the hStemModule and early EBs: NES = 1.686 and FDR = 0 for H1-derived EBs and NES = 1.667 and FDR = 0 for H9-derived EBs (Table 3 and Additional File 5). These results clearly demonstrate the discriminative power of the stemness-relevant module in distinguishing cultured cell types of various stem potentials.

**Table 1:** GSEA of the hStemModule for the stem cell matrix.

Cell types^1^	NES^2^	FDR^3^	Correlation^3^
ePSC	2.132	0.000	Positive**
tPSC	1.899	0.000	Positive**
iPSC	2.658	0.000	Positive**
ePSC_NSC	-1.909	0.000	Negative**
tPSC_Nlin	-0.820	0.888	Null
fNSPC	-2.478	0.000	Negative**
HANSE	-1.843	0.000	Negative**
BM_MSC	-1.754	0.000	Negative**
HUVECS	-2.216	0.000	Negative**
ePSC_EB	2.020	0.000	Positive**

^1^ePSC = embryonic pluripotent stem cells, tPSC = teratocarcinoma pluripotent stem cells, iPSC = induced pluripotent stem cells, ePSC_NSC = embryonic pluripotent stem cell-derived neural stem cells, tPSC_Nlin = teratocarcinoma pluripotent stem cells differentiated into dopaminergic neural lineage, fNSPC = fetal neural stem cell or primary fetal neural precursor cells, HANSE = adult surgery neural precursors, BM_MSC = bone marrow mesenchymal stem cells, HUVECS = umbilical vein endothelial cells, ePSC_EB = embryonic pluripotent stem cell-derived embryoid bodies; ^2 ^(+) NES for positive correlation, (-) NES for negative correlation; ^3^Significance for correlation: *FDR < 0.05, **FDR < 0.01, and 'Null' for no significant correlation.

**Table 2:** GSEA of the hStemModule based on pair-wise comparisons among pluripotent cell types of different origins.

Pair-wise comparisons^1^	NES^2^	FDR^3^	Correlation^3^
iPSC *vs*. ePSC	1.752	0.000	Positive**
iPSC *vs*. tPSC	1.519	0.003	Positive**
tPSC *vs*. ePSC	-0.518	1.000	Null

^1^ePSC = embryonic pluripotent stem cells, tPSC = teratocarcinoma pluripotent stem cells, iPSC = induced pluripotent stem cells; ^2 ^(+) NES for positive correlation, (-) NES for negative correlation; ^3^Significance for correlation: **FDR < 0.01, and 'Null' for no significant association.

**Table 3:** GSEA of the hStemModule for ESC (H1 and H9)-derived EBs.

Cell types^1^	NES^2^	FDR^3^	Correlation^3^
H1_ESC	1.389	0.010	Positive*
H1_EB	1.686	0.000	Positive**
H9_ESC	1.966	0.000	Positive**
H9_EB	1.667	0.000	Positive**

^1^H1_ESC = embryonic stem cell line H1, H1_EB = H1-derived embryoid bodies, H9_ESC = embryonic stem cell line H9, H9_EB = H9-derived embryoid bodies; ^2 ^(+) NES for positive correlation, (-) NES for negative correlation; ^3^Significance for correlation: *FDR < 0.05, **FDR < 0.01.

### The hDiffModule is seemingly able to predict differences in the differentiation potential among pluripotent cells of different origins, but not among differentiated cells of different types

Next, we used the stem cell matrix to examine whether the hDiffModule could be used to evaluate the differentiation potential among different cell types. Our previous work [20] showed that the hDiffModule is largely composed of differentiation-associated genes that are regulated during early human organogenesis. Because those genes are under-expressed in hESCs (i.e., are part of the consensus differentiation gene list defined in [27]), the hDiffModule is expected to negatively correlate with hESCs. Indeed, we observed a significant negative correlation between this module and both ePSCs (NES = -2.234; FDR = 0) and embryonal carcinomas, or tPSCs (NES = -1.490; FDR = 0), but did not observe a correlation between this module and most of differentiated cell types (Table 4; see Discussion). We unexpectedly found that the DiffModule was positively correlated with the iPSCs, the pluripotent cells of non-embryonic origins (NES = 1.373; FDR = 0.029). Consistent with this result, we also found a significant positive correlation between the hDiffModule and iPSCs *vs*. ePSCs (NES = 2.434; FDR = 0) and iPSCs *vs*. tPSCs (NES = 1.847; FDR = 0) (Table 5; see Discussion). Similar to the hStemModule, the hDiffModule was also positively correlated with ePSC_EB (NES = 2.793; FDR = 0) (Table 4); this observation was repeated with a separate dataset (Table 6). Notably, *in vitro *EB differentiation models consistently showed a positive correlation with the hDiffModule, the hStemModule, and the hORGNet made up by these two modules (Additional Files 3 and 5). This suggests the possibility of further characterizing relationships between the developing embryo and the *in vitro *differentiation models that are intended to mimic *in vivo *events.

**Table 4:** GSEA of the hDiffModule for the stem cell matrix

Cell types^1^	NES^2^	FDR^3^	Correlation^3^
ePSC	-2.234	0.000	Negative**
tPSC	-1.490	0.009	Negative**
iPSC	1.373	0.029	Positive*
ePSC_NSC	1.232	0.092	Null
tPSC_Nlin	1.194	0.255	Null
fNSPC	-1.768	0.000	Negative**
HANSE	-1.170	0.111	Null
BM_MSC	1.204	0.130	Null
HUVECS	-1.307	0.041	Negative*
ePSC_EB	2.793	0.000	Positive**

^1,2,3^The same as in Table 1.

**Table 5:** GSEA of the hDiffModule based on pair-wise comparisons among pluripotent cell types of different origins.

Pair-wise comparisons^1^	NES^2^	FDR^3^	Correlation^3^
iPSC *vs*. ePSC	2.434	0.000	Positive**
iPSC *vs*. tPSC	1.847	0.000	Positive**
tPSC *vs*. ePSC	1.085	0.556	Null

^1,2,3^The same as in Table 2.

**Table 6:** GSEA of the hDiffModule for ESC (H1 and H9)-derived EBs.

Cell types^1^	NES^2^	FDR^3^	Correlation^3^
H1_ESC	-2.412	0.000	Negative**
H1_EB	1.608	0.000	Positive**
H9_ESC	-2.072	0.000	Negative**
H9_EB	1.833	0.000	Positive**

^1,2,3^The same as in Table 3.

### The hORGNet-based characterization of relationships between early human organogenesis *in vivo *and hESC-derived EBs *in vitro*

**Figure 3:**
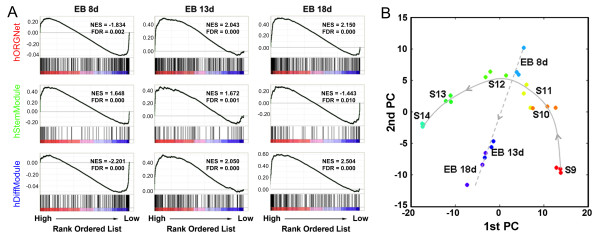
**The hORGNet-based characterization of the relationships between early human organogenesis and an SHhES1-derived EB model**. **(A) **GSEA of the hORGNet (in red) and its two modules, the hStemModule (in green) and hDiffModule (in blue) for time-course transcriptome data from SHhES1-derived EBs at days 8, 13 and 18. Each graph shows the distribution pattern of a gene set over the ranked gent list, together with statistics report (i.e., NES and FDR). **(B) **The PCA of the expression matrix of genes in the hORGNet across the SHhES1-relevant EB models and early human organogenesis. The solid line denotes the *in vivo *developmental trajectory during early human organogenesis, and the dotted line indicates the sustained differentiation process in the SHhES1-derived, *in vitro *EB model.

To further explore the usefulness of the hORGNet (and its two modules) in characterizing relationships between early human organogenesis *in vivo *(S19-S14) and the EB models *in vitro*, we used a time-course of transcriptome data from SHhES1-derived EBs at days 8, 13 and 18 [39] to perform GSEA of the hORGNet and its two modules (Figure 3A). First, we found that the 8-day EB was comparable to S11 (Additional File 6); both were positively correlated with the hStemModule and negatively correlated with the hDiffModule. This is consistent with the timing of the *in vitro *model, which mimics complex *in vivo *events. Second, a positive correlation with the hDiffModule was observed for the 13- and 18-day EBs, which probably reflects the sustained differentiation *in vitro *(the bottom in Figure 3A). The hStemModule experiences a shift from the positive correlation seen in the 8-day EB (NES = 1.648; FDR = 0) to the negative correlation seen in the 18-day EB (NES = -1.443; FDR = 0.010), indicating the loss of the stem potential (the middle in Figure 3A). Third, the GSEA results with respect to the hORGNet showed a tendency towards an increased correlation between the hORGNet and the *in vitro *EB model, partially supporting the idea of sustained differentiation in this *in vitro *EB model (the top in Figure 3A). Therefore, the GSEA analyses suggest a resemblance between the 8-day EB and S11 with regard to both the stem and differentiation potentials, and also suggest that the sustained differentiation *in vitro *in 13- and 18-day EBs could explain their lack of correspondence to any embryonic stage after S11 (see Discussion for details). To vividly display these relationships between the early human organogenesis *in vivo *and this EB model *in vitro*, we performed a principle component analysis (PCA) on the expression matrix of the member genes in the hORGNet during early human organogenesis and during EB differentiation. As illustrated in Figure 3B, two distinct trajectories were revealed, one representing the developmental trajectory *in vivo *during early human organogenesis and the other representing the sustained differentiation *in vitro *in the SHhES1-derived EB model. The positions along each of the trajectories probably reflect the developmental nature of the embryos and the sustained, differentiating nature of the EB model, respectively. These two different trajectories clearly show considerable differences between the developing embryos *in vivo *and the EB cultures over time *in vitro*.

## Discussion

### The different behaviors of the two modules are consistent with the very nature of human embryogenesis

During embryogenesis in humans, early embryonic cells progressively confine their lineage commitment by changing their developmental potential, i.e., their ability to develop into multiple distinct cell types [13]. Cell fate specification during development was first described by *C. Waddington *as the epigenetic landscape [43], which has recently gained popularity with the advent of cell reprogramming [44-47]. In addition to the Waddington landscape, embryonic cells being committed to descendants can also be viewed as a result of a Yin-Yang-like crosstalk between two key aspects of the developmental potential: the stem potential and the differentiation potential. The former is assumed to maintain the stemness properties, while the latter is crucial for specifying the proper differentiation. Together, they act together in harmony to ensure the successful implementation of embryogenesis. The two modules (i.e., the hStemModule and the hDiffModule) in the hORGNet might meet the needs of these two respective roles. The GSEA analysis in Figure 2 shows that the expression patterns of genes in these two modules correlate well with our current knowledge about the development potential of early embryonic cells: a gradual loss of stemness and a concomitant diversity of cell types. Therefore, it is logical to speculate that the monotonous behavior of the hStemModule is the necessary outcome of the gradual loss of the stemness during the embryogenesis. The dynamic changes of the hDiffModule, however, could be required for proper differentiation in a stage-specific and context-dependent manner. The different behaviors of these two modules are the biological basis of their utility, as demonstrated in this study, for distinguishing various cell types (Tables 1, 2, 3, 4, 5 and 6) and characterizing the relationships between embryogenesis *in vivo *and differentiation models *in vitro *(Figure 3).

### The two modules differ in their power to distinguish cultured cells of different types

Choosing a single, yet comprehensive transcriptome dataset (called the stem cell matrix [37]) of various cultured cells as an input may reduce the potential technical biases that could otherwise be introduced when using different types of detection methods from different labs. Processing the stem cell matrix revealed the differences in the discriminative power of the two modules. The hStemModule consistently distinguished cell types of various stem potentials (Table 1). In contrast, the hDiffModule appeared to be unable to distinguish differentiated cells of different types (Table 4), although it provided useful clues for pluripotent cells of different origins (for a detailed discussion, see the next subsection). Their differences in discriminative power can be partially explained by the expression patterns of their member genes in hESCs compared to many differentiated cell types [27]. In our previous work [20], we have showed that the hStemModule is enriched for stemness-relevant genes that are consistently over-expressed in hESCs, while the hDiffModule contains differentiation-relevant genes that are consistently under-expressed in hESCs. In other words, we know for sure that the genes in the hStemModule should be consistently expressed in hESCs. However, the genes in the hDiffModule may be expressed in one or more types of differentiated cells that we cannot identify with certainty, and the hDiffModule itself is therefore not informative regarding differentiated cells of different types.

### Differences and similarities of the two modules in distinguishing pluripotent cells of different origins

Pluripotent cells were first isolated from embryonic sources, such as ePSCs from the inner cell mass of the blastocyst [48] and tPSCs from embryonal carcinoma [49]. As a new source of pluripotent cells, iPSCs are generated from non-pluripotent cells (typically somatic cells) that are genetically reprogrammed to an ePSC-like state [50-53]. Initially, iPSCs were thought to be quite similar to their embryonic counterparts, but recent studies have suggested substantial differences between them at both the gene expression [54] and the epigenetic levels [55]. In this study, we showed that the hStemModule positively correlates with all types of pluripotent cells (Table 1), whereas the hDiffModule is negatively correlated with the pluripotent cells of embryonic origins other than iPSCs (Table 4). This difference may be meaningful. In terms of the stem potential, all pluripotent cells should share the characteristics of pluripotency. However, with regard to the differentiation potential, the pluripotent cells of embryonic origins completely repress the expression of differentiation-associated genes, while iPSCs derived from the differentiated cells may inevitably retain an imprint from their origins despite being reprogrammed to a fully pluripotent state. Apart from this difference, we also observed the similarities between the two modules when comparing pluripotent cells of different origins. Compared with the pluripotent cells of embryonic origins (ePSCs and tPSCs), iPSCs showed a significant positive correlation with each of the two modules (Tables 2 and 4). Although the exact implication remains unclear, this may reflect the unique nature of iPSCs; their stemness- and differentiation-contexts may be more similar to those of the hStemModule and the hDiffModule than those of pluripotent cells of the embryonic origins. Future studies will clarify these observations in a wet experimental setting.

### Recommended circumstances for using the two modules

In addition to the above situation involving pluripotent cells of different origins, we suggest that the following *a priori *knowledge will be indispensable for using two modules as a monitor of the stem potential *versus *differentiation potential, especially for the hDiffModule (and the hORGNet). Owing to the stage-specific and context-dependent nature of the hDiffModule (Figure 2), it is only valid when the comparisons are constrained to the S9-S14 developmental window or the equivalent *in vitro *differentiation processes (such as the EB models in Figure 3) that recapitulate the *in vivo *cues of this developmental window. The stage-specific nature of the hDiffModule (and thus the hORGNet) does not necessarily mean that it is unsuitable for characterizing the S9-S14 or the equivalent models. Genes in the hDiffModule show both reduced and increased expression patterns from S9 to S14 (see in our previous study [20]), and the positive or negative correlation from the GSEA analysis implies the extent of expression changes required for proper differentiation at each stage. The stage-specific expression profiles of the hDiffModule-containing genes are indicative of each stage, and their correlations (no matter being positive or negative) all have statistical significance as shown in Figure 2A. Together with the hStemModule, the hDiffModule gives the hORGNet as a specific signature for each stage during early human organogenesis from S9 to S14. Additionally, it raises the possibility of using the hORGNet to understand relationships between early human organogenesis *in vivo *and EB models *in vitro*.

### Implications of the differences between the *in vivo *and *in vitro *developmental trajectories captured by the hORGNet

The observations of (i) the resemblance between 8-day EB and S11 and (ii) the divergence of 13- and 18-day EBs away from the subsequent developmental stages (i.e., S12-S14), have several implications. First, hESCs differentiated *in vitro *into EBs can mimic events that occur *in vivo *both before and after the embryonic implantation [56,57], even extending to S11 at least in terms of the stem and differentiation potential. Second, the prolonged differentiation in culture raises concerns over the limitations of *in vitro *EB models. The EB at days 13 and 18 tended to be positively correlated with the hDiffModule, which is probably due to the sustained expression of the same subsets of differentiation-associated genes that make up the hDiffModule. However, in *in vivo *embryos, different subsets of differentiation-associated genes from the hDiffModule were expressed at each of the different stages, even though the overall correlations between the hDiffModule and stages S13/S14 also remained significantly positive. This selective expression of hDiffModule genes in the developing embryos *in vivo *and the sustained expression of hDiffModule genes in EBs cultured *in vitro *could explain the two different trajectories as revealed in Figure 3B. Finally, the two overlapping, yet different trajectories observed in this study will warrant the use of this integrated network and its two modules in future studies on human embryogenesis, both *in vivo *and *in vitro*. This network will be particularly useful for those studies that focus on evaluating the stem potential *versus *the differentiation potential.

## Conclusions

Using a previously proposed integrated network (hORGNet) and its two modules, the stemness-relevant module (hStemModule) and the differentiation-relevant module (hDiffModule), we illustrate its utility by analyzing transcriptome data from a wide variety of developmental contexts (Figure 1). This analysis provides new insights into early human development, both *in vivo *and *in vitro *(Figures 2 and 3; Tables 1, 2, 3, 4, 5 and 6). These advances add an additional dimension to network applications. We strongly recommend the use of this network and its two modules for the circumstances (i) when pluripotent cell types of different origins are involved and (ii) when the comparisons are constrained to the in *vivo *embryos during early human organogenesis or to the equivalent *in vitro *differentiation processes. As the transcriptome data coverage for human embryos improves, we anticipate that even more precise relationships will be revealed using similar network-based comparative transcriptome analyses.

## Methods

### A putative molecular interaction network during early human organogenesis

In our recent work [20], we performed a transcriptome analysis of human embryos from Carnegie stages 9 (S9) to 14 (S14), which covers the first third of organogenesis. Further integration of this expression data with interaction information allowed us to identify a putative molecular interaction network that coordinates early human organogenesis (termed hORGNet). A preliminary analysis revealed that the hORGNet is composed of two relatively distinct modules, a stemness-relevant module (hStemModule) and a differentiation-relevant module (hDiffModule). Here, we further evaluate the utility of this hypothetical network and its two modules for characterizing the stem potential *versus *the differentiation potential in various developmental contexts (see below).

### Sources of transcriptome data from a variety of developmental contexts

The stem cell matrix [37] was obtained from NCBI GEO (GSE11508). It contains transcriptome data from the cultured stem cells in the context of a wide variety of pluripotent, multipotent and differentiated cell types. Based on the published cluster results of core dataset samples (further restricted by sample information, such as source tissue, cell type, differentiation state and lineage of the cells), 136 out of the 219 samples were extracted and annotated as belonging to one of 10 clusters. Samples within each cluster displayed similar expression profiles as revealed by a component plane presentation integrated self-organizing map (CPP-SOM) [58,59]. These clusters, each associated with biological- and profile-similar characteristics, included embryonic pluripotent stem cells (ePSC), induced pluripotent stem cells (iPSC), teratocarcinoma pluripotent stem cells (tPSC), embryonic pluripotent stem cell-derived neural stem cells (ePSC_NSC), teratocarcinoma pluripotent stem cells differentiated into dopaminergic neural lineage (tPSC_Nlin), fetal neural stem cell or primary fetal neural precursor cells (fNSPC), adult surgery neural precursors (HANSE), bone marrow mesenchymal stem cells (BM_MSC), umbilical vein endothelial cells (HUVECS) and embryonic pluripotent stem cell-derived embryoid bodies (ePSC_EBs).

Transcriptome data for early stage EBs (3.5 days) derived from two human ESC lines (H1 and H9) were obtained from a published study [38], and a time course transcriptome dataset from the SHhES1-derived EBs at days 8, 13 and 18 was obtained from a previously published report [39]. Two genome-wide expression datasets for human embryos at six successive time periods (days 20-32) [20] and at six interval-longer time points (weeks 4-9) [21] were obtained from NCBI GEO using the accession numbers GSE1887 and GSE15744, respectively.

### Gene set enrichment analysis (GSEA) of the hORGNet and its two modules

GSEA [60] is a computational method for determining whether an *a priori *defined set of genes (e.g., those genes in the hORGNet) shows statistically significant, concordant differences between two biological states (e.g., one embryonic stage compared to the average of all human embryo stages). We used GSEAPreranked to determine the degree to which genes in the hORGNet (and its two modules, hStemModule and hDiffModule) were overrepresented at the top or bottom of a predefined list of ranked genes. The ranked lists of genes were predefined according to transcriptome data sources as mentioned above in the previous subsection. These gene lists were ranked by means of LIMMA supervised analysis [41], which uses linear models and empirical Bayes methods to assess differential expression. GSEA calculates an enrichment score (ES) to reflect the enrichment of a gene set at the top (a positive ES) or bottom (a negative ES) of a ranked list of genes. Accounting for differences in the gene set size, GSEA also reports a normalized enrichment score (NES) for comparing results across different gene sets. The significance of the enrichment associated with each NES can be assessed by estimating the false discovery rate (FDR [61]). The detailed explanations for these GSEA statistics can be found in the original paper [40].

For the stem cell matrix, LIMMA supervised analysis was used to determine the ranked gene lists between the assigned cluster and the remaining clusters (or between the assigned cluster and another cluster), followed by GSEA of the hStemModule and the hDiffModule. Similar analysis was also applied to transcriptome data of H1/H9-derived EBs. Regarding human embryos at stages S9-S14, SHhES1-derived EB at days 8, 13 and 18, and human embryos at weeks 4-9, LIMMA was applied to predefine the ranked gene lists between each time point against the average of all time points. GSEA results (i.e., NES and FDR) are detailed in Additional Files 3, 4, 5 and 6. An FDR of 0.05 or lower was accepted as indicating statistical significance for NES (positive or negative).

## Abbreviations

hORGNet: human organogenesis network; hStemModule: stemness-relevant module; hDiffModule: differentiation-relevant module; hESCs: human embryonic stem cells; EBs: embryoid bodies; GSEA: gene set enrichment analysis; NES: normalized enrichment score; FDR: false discovery rate; LIMMA: linear models for microarray data; PCA: principle component analysis; CPP-SOM: component plane presentation integrated self-organizing map; ePSC: embryonic pluripotent stem cells; iPSC: induced pluripotent stem cells; tPSC: teratocarcinoma pluripotent stem cells; ePSC_NSC: embryonic pluripotent stem cell-derived neural stem cells; tPSC_Nlin: teratocarcinoma pluripotent stem cells differentiated into dopaminergic neural lineage; fNSPC: fetal neural stem cell or primary fetal neural precursor cells; HANSE: adult surgery neural precursors; BM_MSC: bone marrow mesenchymal stem cells; HUVECS: umbilical vein endothelial cells; ePSC_EB: embryonic pluripotent stem cell-derived embryoid bodies.

## Competing interests

The authors have declared that no competing interests exist.

## Authors' contributions

HF conceived and designed the study, carried out the data analysis and interpretation, and drafted and revised the manuscript. WJ contributed to the data analysis and revised the manuscript. YY contributed to materials and participated in the design of the study. YJ contributed to materials and helped the data interpretation. JZ conceived the study, participated in its coordination and helped to draft the manuscript. KKW designed and coordinated the study, interpreted the results, and drafted and revised the manuscript. All authors read and approved the final manuscript.

## Supplementary Material

Additional file 1 **The content of hORGNet**. This table lists genes contained in the hORGNet and its two modules, a stemness-relevant module (hStemModule) and a differentiation-relevant module (hDiffModule). Click here for file

Additional file 2 **CPP-SOM of the stem cell matrix**. Out of the 219 samples in the stem cell matrix, 136 were extracted according to published cluster results and sample information (e.g., source tissue, cell type, differentiation state and lineage of the cells). They were grouped into 10 clusters, each associated with biological- and profile-similar characteristics. The transcriptome profiles are visualized by Component plane presentation integrated self-organizing map (CPP-SOM). Each presentation illustrates a sample-specific transcriptome map, in which all of the up-regulated (represented by neurons in red), down-regulated (represented by neurons in blue) and moderately regulated (represented by neurons in yellow and green) genes are well delineated. All the presentations are linked by positions. The colours bar stands for expression values (log ratio with base 2), with brighter colours denoting the higher values. Click here for file

Additional file 3 **GSEA using the stem cell matrix**. GSEA of the hORGNet and its two modules (hStemModule and hDiffModule) using transcriptome data from the stem cell matrix. Click here for file

Additional file 4 **GSEA for comparing pluripotent cells of different origins**. GSEA of the hORGNet and its two modules (hStemModule and hDiffModule) was performed for pair-wise comparisons between embryonic pluripotent stem cells (ePSC), teratocarcinoma pluripotent stem cells (tPSC), and induced pluripotent stem cells (iPSC). Notably, when compared to ePSCs and tPSCs, iPSCs are more likely to be associated with the hStemModule (in terms of the stemness potential) and the hDiffModule (in terms of the differentiation potential), respectively. Click here for file

Additional file 5 **GSEA using transcriptome data of early stage EBs**. GSEA of the hORGNet and its two modules (hStemModule and hDiffModule) was performed using transcriptome data of early stage EBs (3.5 days) derived from two human ESC lines (H1 and H9). Notably, GSEA results indicated significant positive correlations between the hStemModule/hDiffModule and early EBs. Click here for file

Additional file 6 **Comparisons of GSEA results**. GSEA results with in vivo early human organogenesis (S11-S14) **(A) **and in vitro EB model (8 d, 13 d and 18 d) **(B) **were compared. Based on NES profiles, 8-day EB is matched to the S11 (framed in pink), which is consistent with the timing of this *in vitro *model that mimics complex *in vivo *events. The expression-based positive correlation between the hDiffModule and 13-day (and 18-day) EB probably reflects the *in vitro *sustained differentiation of the *in vivo *S11, which is further inferred from the tendency toward increased correlation between the hORGNet and the *in vitro *EB model. Click here for file
