# Supplementary figures and images for: An organogenesis network-based comparative transcriptome analysis for understanding early human development in vivo and in vitro
Source: BMC Syst Biol. 2011 Jul 6;5:108. doi: 10.1186/1752-0509-5-108 (PMC3141417; doi:10.1186/1752-0509-5-108)

ePSC (n = 17)

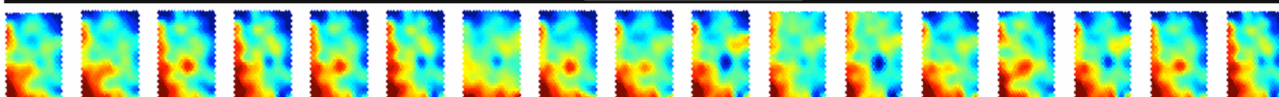

iPSC (n = 3)

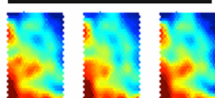

tPSC (n = 5)

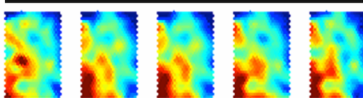

ePSC\_NSC (n = 17)

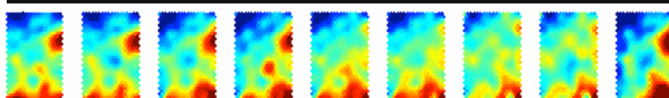

tPSC\_Nlin (n = 17)

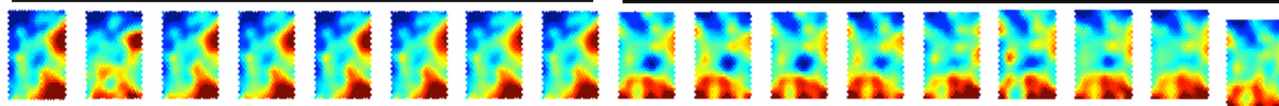

fNSPC (n = 23)

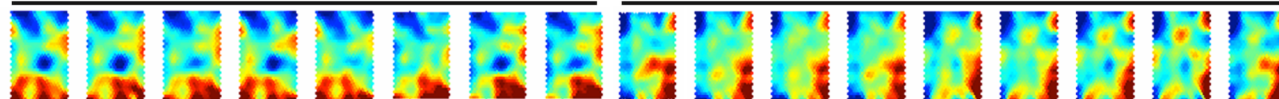

HANSE (n = 32)

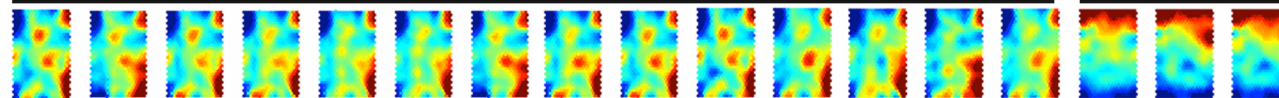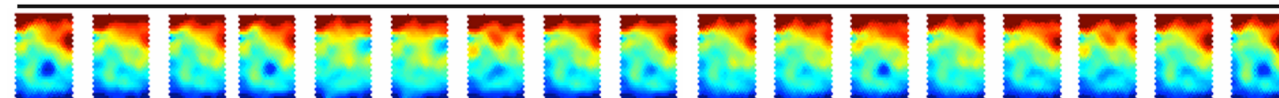

BM\_MSC (n = 10)

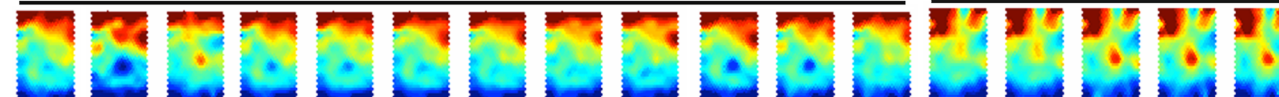

HUVECS (n = 6)

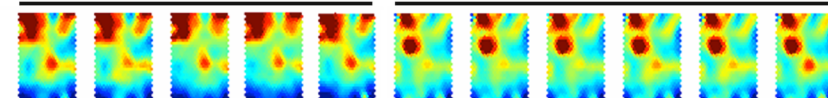

ePSC\_EB (n = 6)

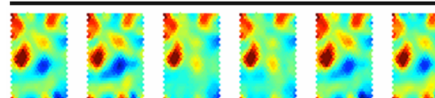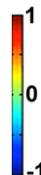

Supplement: Additional file 2 — CPP-SOM of the stem cell matrix. Out of the 219 samples in the stem cell matrix, 136 were extracted according to published cluster results and sample information (e.g., source tissue, cell type, differentiation state and lineage of the cells). They were grouped into 10 clusters, each associated with biological- and profile-similar characteristics. The transcriptome profiles are visualized by Component plane presentation integrated self-organizing map (CPP-SOM). Each presentation illustrates a sample-specific transcriptome map, in which all of the up-regulated (represented by neurons in red), down-regulated (represented by neurons in blue) and moderately regulated (represented by neurons in yellow and green) genes are well delineated. All the presentations are linked by positions. The colours bar stands for expression values (log ratio with base 2), with brighter colours denoting the higher values. [file 1752-0509-5-108-S2.PDF]

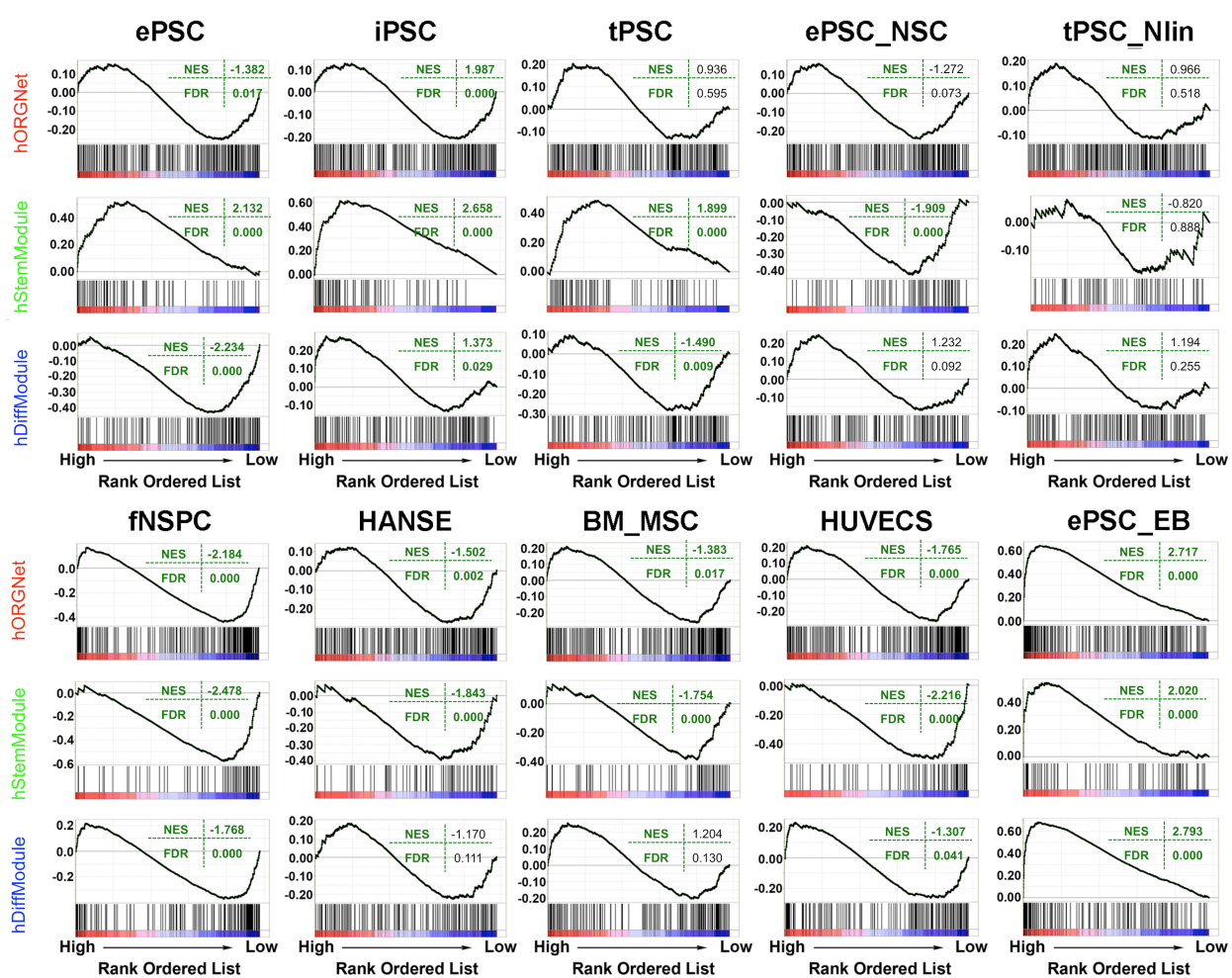

Supplement: Additional file 3 — GSEA using the stem cell matrix. GSEA of the hORGNet and its two modules (hStemModule and hDiffModule) using transcriptome data from the stem cell matrix. [file 1752-0509-5-108-S3.PDF]

A

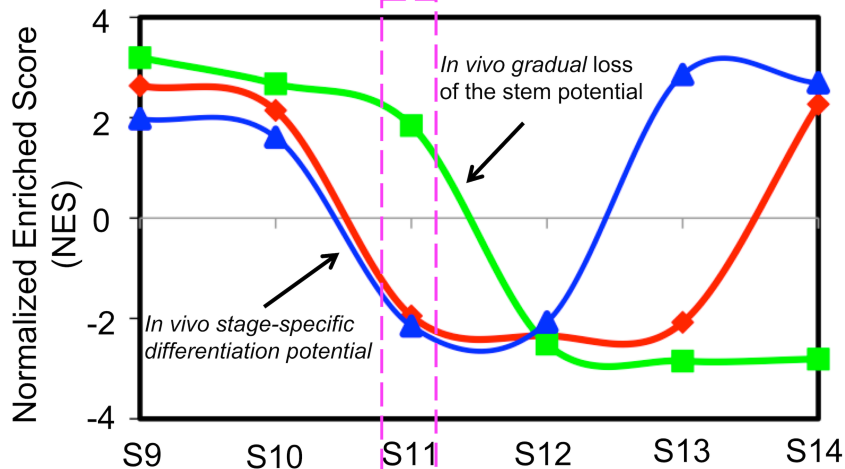

B

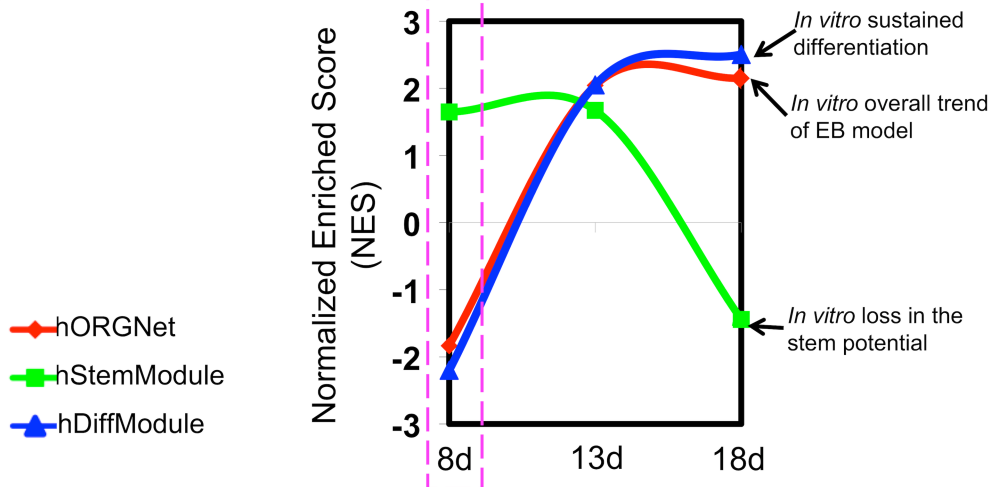

Supplement: Additional file 6 — Comparisons of GSEA results. GSEA results with in vivo early human organogenesis (S11-S14) (A) and in vitro EB model (8 d, 13 d and 18 d) (B) were compared. Based on NES profiles, 8-day EB is matched to the S11 (framed in pink), which is consistent with the timing of this in vitro model that mimics complex in vivo events. The expression-based positive correlation between the hDiffModule and 13-day (and 18-day) EB probably reflects the in vitro sustained differentiation of the in vivo S11, which is further inferred from the tendency toward increased correlation between the hORGNet and the in vitro EB model. [file 1752-0509-5-108-S6.PDF]
