# Supplementary material for: An organogenesis network-based comparative transcriptome analysis for understanding early human development in vivo and in vitro
Source: BMC Syst Biol. 2011 Jul 6;5:108. doi: 10.1186/1752-0509-5-108 (PMC3141417; doi:10.1186/1752-0509-5-108)
Supplement: Additional file 4 — GSEA for comparing pluripotent cells of different origins. GSEA of the hORGNet and its two modules (hStemModule and hDiffModule) was performed for pair-wise comparisons between embryonic pluripotent stem cells (ePSC), teratocarcinoma pluripotent stem cells (tPSC), and induced pluripotent stem cells (iPSC). Notably, when compared to ePSCs and tPSCs, iPSCs are more likely to be associated with the hStemModule (in terms of the stemness potential) and the hDiffModule (in terms of the differentiation potential), respectively. [file 1752-0509-5-108-S4.PDF]

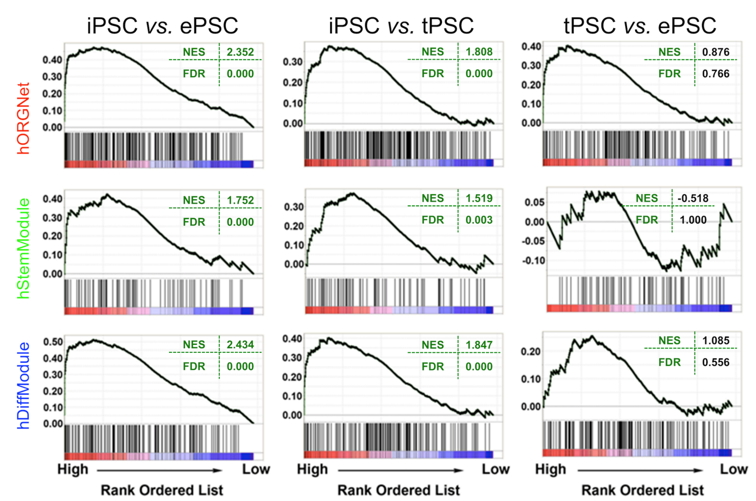
iPSC vs. ePSC
iPSC vs. tPSC
tPSC vs. ePSC
hORGNet
hStemModule
hDiffModule
NES 2.352
FDR 0.000
NES 1.808
FDR 0.000
NES 0.876
FDR 0.766
NES 1.752
FDR 0.000
NES 1.519
FDR 0.003
NES -0.518
FDR 1.000
NES 2.434
FDR 0.000
NES 1.847
FDR 0.000
NES 1.085
FDR 0.556
High
Low
Rank Ordered List
