# Supplementary material for: An organogenesis network-based comparative transcriptome analysis for understanding early human development in vivo and in vitro
Source: BMC Syst Biol. 2011 Jul 6;5:108. doi: 10.1186/1752-0509-5-108 (PMC3141417; doi:10.1186/1752-0509-5-108)
Supplement: Additional file 5 — GSEA using transcriptome data of early stage EBs. GSEA of the hORGNet and its two modules (hStemModule and hDiffModule) was performed using transcriptome data of early stage EBs (3.5 days) derived from two human ESC lines (H1 and H9). Notably, GSEA results indicated significant positive correlations between the hStemModule/hDiffModule and early EBs. [file 1752-0509-5-108-S5.PDF]

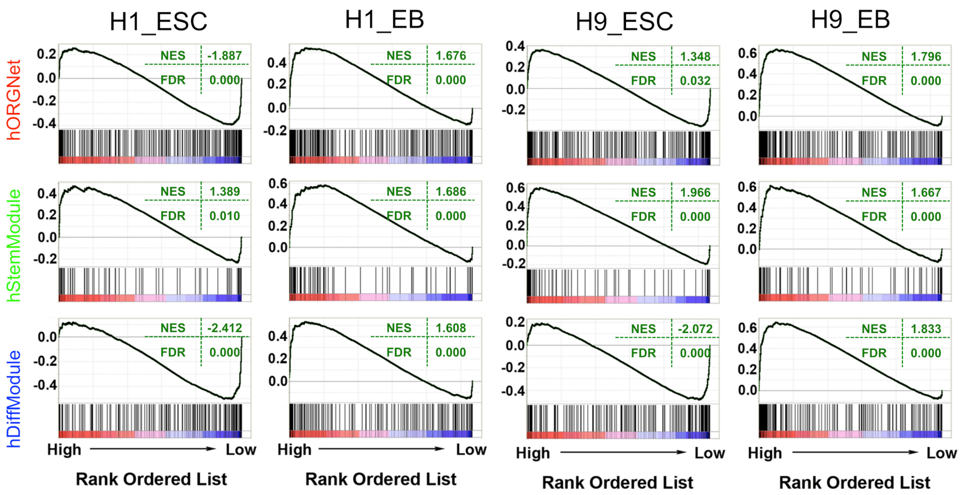

H1_ESC
H1_EB
H9_ESC
H9_EB
hORGNet
hStemModule
hDiffModule
NES -1.887 FDR 0.000
NES 1.676 FDR 0.000
NES 1.348 FDR 0.032
NES 1.796 FDR 0.000
NES 1.389 FDR 0.010
NES 1.686 FDR 0.000
NES 1.966 FDR 0.000
NES 1.667 FDR 0.000
NES -2.412 FDR 0.000
NES 1.608 FDR 0.000
NES -2.072 FDR 0.000
NES 1.833 FDR 0.000
High
Low
Rank Ordered List
